# Searching for constituents from plants in geographically characterized areas, Egypt, Madagascar, and Okinawa

**DOI:** 10.1007/s11418-022-01638-x

**Published:** 2022-08-12

**Authors:** Sachiko Sugimoto

**Affiliations:** Department of Pharmacognosy, Graduate School of Biomedical and Health Sciences, Hiroshima University, 1-2-3 Kasumi, Minami-ku, Hiroshima, 734-8553 Japan

**Keywords:** *Ixora undulata*, *Onopordum alexandrinum*, *Entada phaseoloides*, *Cinnamosma fragrans*, *Grevillea robusta*, *Dodonaea viscosa*

## Abstract

Secondary metabolites may not be produced under some conditions, and in most cases, their function and significance in the producing organisms is unknown. Conversely, there are some that are produced for readily understood reasons, for example, toxic substances as defensive substances against invaders, or volatile substances that attract other species of organisms. These secondary metabolites also contribute to our health. However, there has not been sufficient research to evaluate them from a pharmacological perspective, and much progress is expected in this area in the future. About 90% of the existing plants have not been studied for their chemical components and biological activities (Kazuki Saito in Bunshun shinsho 1119, pp. 119–126. ISBN 978-4-16-661119-5, 2017). On this basis, we have been searching for the constituents of unknown plants, and whose constituents have not been studied extensively. In this paper, the authors have reviewed some of their previous searching for constituents from plants in geographically characterized areas, Egypt, Madagascar, and Okinawa.

## Introduction

Today, research on the discovery of biologically active substances from natural products is being actively conducted in many countries around the world, greatly contributing to humanity through the development of lead compounds for pharmaceuticals and pharmacological reagents that exhibit a specific mechanism of action. The researchers are investigating various natural medicines, marine organisms, microorganisms, tropical plants and animals, and so on. Among them, natural products, such as Japanese and Chinese herbal medicines, have been handed down to the present generation through experiential knowledge by application to humans since ancient times. These herbal medicines are deemed as pharmaceutical materials with proven efficacy, and some of their components can become lead pharmaceutical compounds. However, many of these natural products have not been examined for their constituents. Thus, it is important to elucidate the active ingredients from natural products and investigate their pharmacological aspects.

The authors have isolated several novel compounds from plants of unknown composition native to Egypt [2, 3], Madagascar [4–6], Thailand [7, 8], and Okinawa [9–12], and determined their chemical structures. We have also found various pharmacological actions of the isolated compounds.

## Egyptian plant constituent exploration

## Isolation of sulfur-containing alkaloids from *Ixora undulata* [1]

**Fig. 1:**
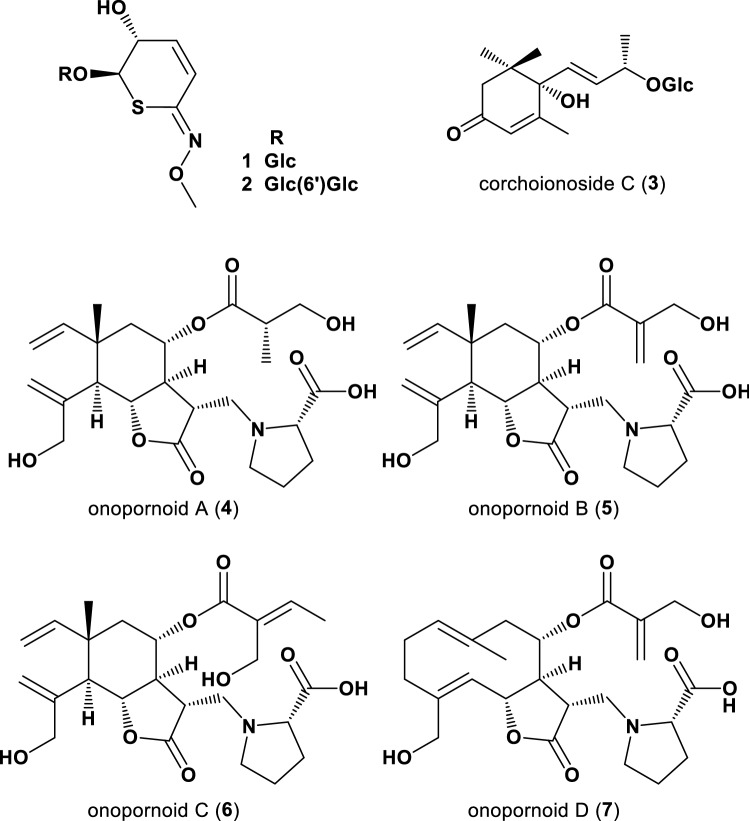
Structures of Isolated Compounds from Egyptian Plants

*Ixora* is a genus in the family Rubiaceae, which contains tropical evergreens and shrubs. Over 400 *Ixora* species exist in tropical Asia, where people widely use it for ornamental and medicinal purpose. *I*. *chinensis*, one of the most common native species found in southern China, has been previously reported that its leaves contain iridoid glucosides [13]. Similarly, *I*. *coccinea*, a dense shrub, which is native to India, is commonly used in traditional medicine [14]. Interestingly, *I. undulata,* which is collected in Egypt, is popularly used in religious ceremonies and as an ornamental plant. However, its constituents are unknown. We isolated a crystalline sulfur-containing alkaloid glycoside and determined its absolute configuration using X-ray crystallographic analysis. 1-(*R*)-phenyl ethanol *β*-gentiobioside (**1**) and 2-methylphenylmethanol *β*-gentiobioside (**2**) have a relatively rare aglycone, which contains three heteroatoms, such as oxygen, nitrogen, and sulfur (Fig. 1). We also found that megastigmane glycosides exhibited glycosylation inhibitory activity. Advanced glycation endproducts (AGEs), which readily form and accumulate with sustained hyperglycemia, contribute to the development of diabetic complications and are considered a potential therapeutic target. Corchoinoside C (**3**) showed strong inhibitory activity toward AGEs formation with an IC_50_ value of 86.0 μM. The inhibitory activity of a positive control, aminoguanidine, was 2.48 mM. Aminoguanidine once entered the phase II clinical trials but was withdrawn due to its side effects [15]. Our results indicate that one megastigmane glucoside was clearly more efficient in inhibiting the formation of AGEs than the positive control. Thus, these data warrant further detailed investigation of these compounds as potential therapeutic agents for diabetic complications and related diseases.

## Isolation of sesquiterpene-amino acid conjugates from *Onopordum alexandrinum* [3]

*Onopordum alexandrinum* Boiss. (family: Asteraceae) is naturally distributed in the state of Israel, the Hashemite Kingdom of Jordan, and Egypt. The consumption of its tuberous roots by the natives of the western Egyptian desert causes hallucination and even death in some cases at high doses [16, 17]. *O. alexandrinum* is a biennial, short-lived perennial plant with coarse, spiny leaves and conspicuous spiny-winged stems. The genus *Onopordum* comprises ~ 50 species, which are distributed across Europe, North Africa, and Southwest Asia. *Onopordum* species have been chemically and biologically studied [18]. Sesquiterpenoids and lignans have been isolated from *O. laconicum* and *O. acanthium*, respectively [18, 19], and cynarine, a quinic acid ester with anti-oxidant activity, was isolated from *O. illyricum* [20]. However, detailed phytochemical investigation of the whole aerial parts of *O. alexandrinum*, including leaves, stems, and flower buds, is yet to be conducted. We isolated four new sesquiterpene-amino acid conjugates, onopornoids A–D (**4**–**7**) (three elemans and one germacrane) (Fig. 1). These amino acids were also identified as L-proline using acid hydrolysis with 1 M HCl followed by HPLC analysis with a chiral detector [21]. Asteraceae plants are rich in sesquiterpenes, but sesquiterpene-amino acid conjugates are unusual.

## Madagascan plants’ constituents’ exploration

## The world’s largest bean: exploring the ingredients of *Entada phaseoloides* [4]

**Fig. 2:**
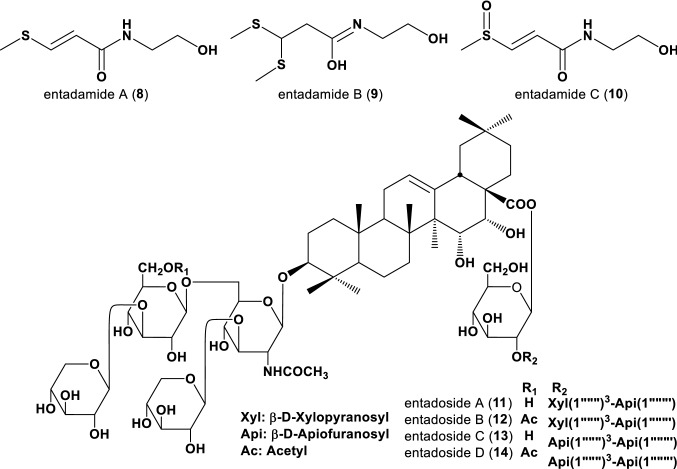
Structures of entadamides (**A**–**C**) (**8**–**10**) and entadosides (**A**–**D**) (**11**–**14**)

*Entada phaseoloides* (L.) Merrill is a liana of the Fabaceae family and is native to the tropical areas. Kernel nuts of *Entada* species possess anti-inflammatory activity [22] and are used as a substitutive of soap due to high content of saponins. A set of unique sulfur-containing amides, entadamides A‒C (**8**–**10**), were isolated from *E*. *phaseoloides* [23–25] along with entadamide A glucoside [26] (Fig. 2). Our study on the constituents of kernel nuts of *E*. *phaseoloides*, collected in Veco Pacca, Madagascar, highlighted four new *N*-acetylglucosamine-containing saponins, named entadosides A‒D (**11**–**14**) (Fig. 2). Compounds **12** and **14** showed strong cytotoxicity against in the human carcinoma cell line, A549 (IC_50_: 10.5 ± 1.9 μM and 17.3 ± 6.6 μM, respectively, whereas other two saponins, **11** and **13**, showed moderate activity (IC_50_: 31.9 ± 3.0 μM and 56.7 ± 11.6 μM, respectively). Acetylation onto 6ʹʹʹ-alcohol remarkably enhanced the activity and as a general trend, xylopyranosides to the 2ʹʹʹʹʹ-position of ester-linked glucose were more effective than apiofuranosides.

## Sesquiterpene lactam obtained from *Cinnamosma fragrans* [5, 6]

**Fig. 3:**
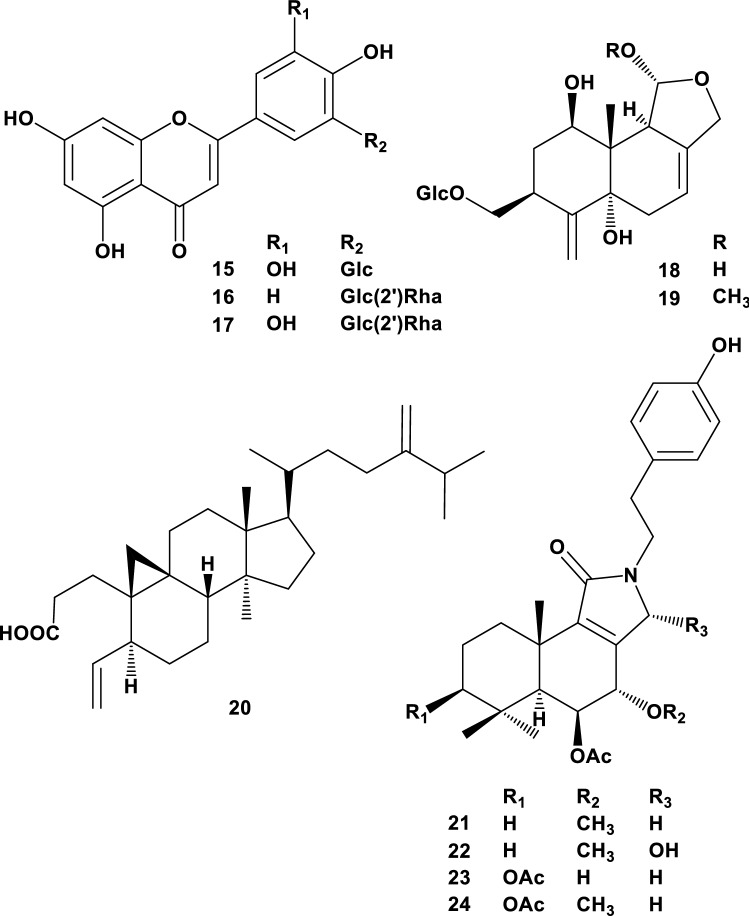
Structures of isolated compounds (**15**–**24**) from *C. fragrans*

*Cinnamosma fragrans* Baillon (Canellaceae) is an endemic plant in the northwestern and east central areas of Madagascar. A decoction of the bark of *C. fragrans* is traditionally used for treating malarial symptoms [27]. *C. fragrans* contains fragrant essential oils, 1,8-cineol and linalool, as antimicrobial agents [28], and the isolation of extremely bitter drimane-type sesquiterpenes has also been previously reported [29–31]. Three C-glycosides (**15**–**17**), two coloratane-type sesquiterpene glycosides (**18**, **19**), one triterpene (**20**), and four drimane-type sesquiterpene lactams (**21**–**24**) were isolated and structurally determined as new compounds from this plant (Fig. 3). Compounds **21**, **22**, and **24**, which have a tyramine residue and a methoxy substituent at position 7, showed anti-multidrug resistance activity and 44.2 ± 3.3, 37.5 ± 2.8, and 56.1 ± 3.4% inhibition at 100 μM, respectively (**24**: IC_50_ = 41.5 ± 3.5 μM). Of these, the drimane-type sesquiterpene lactam was unusual structure. Sesquiterpene lactams have rarely been found in nature; the ones found include cespilactam A from a soft coral, *Cespitularia hypotentaculata* [32], and curdionolide C from *Curcuma wenyujin* (Zingiberaceae) [33]. Nitrogen atoms in these sesquiterpenes result in imperfect-type alkaloids. Haumanamide (from *Spongia* sp.) is the only known isolated diterpene lactam conjugated with phenethylamine. [34].

## Okinawan plant constituent exploration

This paper introduces two species of Okinawan plants from the studies we have conducted on their constituents.

## Isolation of arbutin derivatives exhibiting inhibitory activity on melanin production from *Grevillea robusta* [9, 10]

**Fig. 4:**
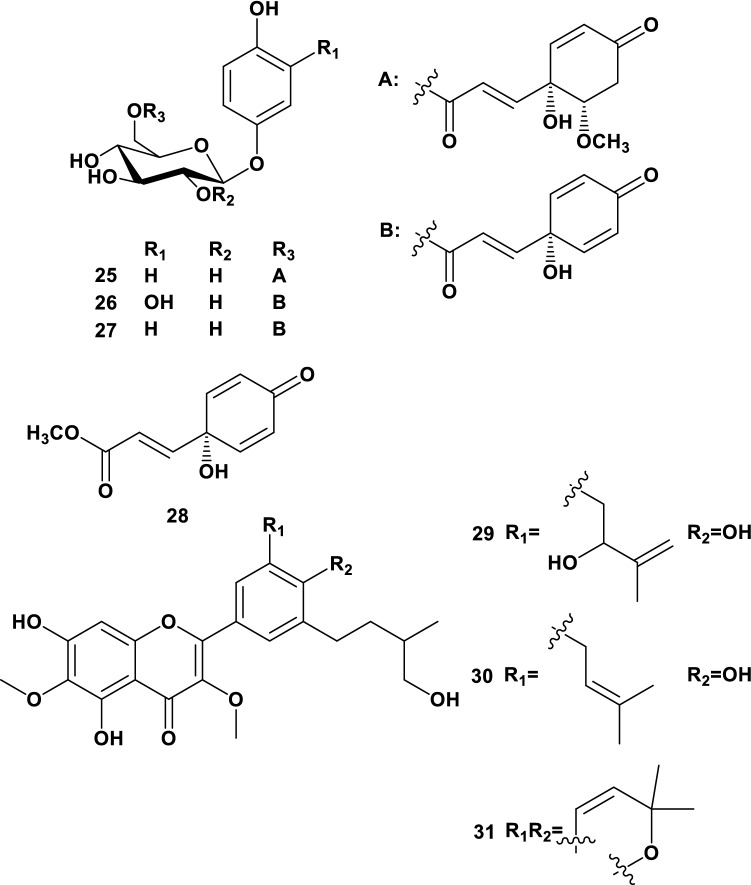
Structures of Isolated Compounds from Okinawan Plants and dodoviscin A (**29**)

*Grevillea robusta*, which belongs to the Proteaceae, originates from subtropical areas of eastern Australia and is planted in Japan for ornamental purposes. It is an evergreen tree between 20 and 35 m in height, with dark green delicately dented bipinnatifid leaves reminiscent of fronds. The leaves are 15–30 cm long with gray–white or rusty undersides. A phytochemical investigation of the same plant, collected in Egypt, has been reported and several phenolic glucosides were isolated [35]. Cytotoxic 5-alkylresorcinol metabolites were also isolated from this plant [36], and a MeOH extract of its timber exhibited potent leishmanicidal activity [37]. Our laboratory has also isolated and reported several 5-alkylresorcinol derivatives from the same plant [38]. Additionally, *G. robusta* was a rich source of arbutin derivatives in our study. The compounds isolated in this study were assayed for their melanogenesis inhibitory activity using mouse melanoma cells (B16). Significant melanogenesis inhibitory activity was observed for some arbutin derivatives using B16 melanoma cells. Then, we further confirmed using a high melanin-producing clone, B16Y24, established in this study. Although B16Y24 is a potent melanin producer, grevilloside O (**26**) and robustaside D (**27**) inhibited melanogenesis moderately, and grevilloside M (**25**) and graviquinone (**28**) possessed potent inhibitory activity toward it (Fig. 4, Table 1). Notably, their strong melanogenesis inhibitory activity showed almost no association with cytotoxicity. Considering the structure and activity relationship, these compounds possessed a common ester moiety, i.e., 3-(1-hydroxy-4-oxocyclohexa-2,5-dien-1-yl) acrylate or (*E*)-3-(1,6-dihydroxy-4-oxocyclohex-2-en-1-yl) acrylate.

**Table 1:** Melanogenesis inhibitory activity

Compound	Melanogenesis IC_50_ (µM)	Cytotoxicity IC_50_ (µM)
**25**	7.5 ± 3.1	> 30
**26**	52.9 ± 2.5^a^	> 100^a^
**27**	20.7 ± 1.8	> 30
**28**	11.3 ± 0.1	> 30
Arbutin	175.1 ± 3.4^a^	> 300^a^

Each value represents the mean ± S.D. for quadruple experiments

^a^The dose was increased up to 100 or 300 µM to determine the IC_50_ values

## Research on the constituents of *Dodonaea viscosa* [12]

*Dodonaea viscosa* Jacquin (family: Sapindaceae) is a small evergreen tree (around 3–5 m in height) that is naturally distributed in Japan (Nansei Islands and Ogasawara Islands), Australia, New Zealand, and other tropical to subtropical regions of the world. It is an oval-shaped tree that branches from the lower section of the aerial part of the plant. Its leaves are glossy green and alternately oblong at all edges. From March to April, it forms short panicles to produce inconspicuous yellow–green flowers. Several parts of *D. viscosa* have been used in traditional medicine to treat several diseases in East Africa. As part of our research to find the constituents of Okinawan plants, we performed a search for the constituents of methanol extract of this plant. We describe the isolation of three new diterpenes and known compounds. Dodoviscin A (**29**) (Fig. 4), a compound isolated from *D. viscosa*, inhibits melanin production [39]. However, a detailed investigation of this plant species is yet to be conducted. Collagen is a major component of the dermis that keeps the skin elastic and firm. On the other hand, collagenase is an enzyme that breaks down the collagen and causes skin aging (e.g., as wrinkles). 5,7,4ʹ-trihydroxy-3ʹ-(4-hydroxy-3-methylbutyl)-5′-(3-methylbut-2-enyl)-3,6-dimethoxyflavone (**30**) showed the most potent collagenase inhibitory activity (IC_50_ = 42.9 ± 6.0 μM), while dodoviscin C (31) showed almost the same activity as the positive control (caffeic acid), IC_50_ = 94.5 ± 17.7 μM, 89.7 ± 4.8 μM, respectively. Similar to, compounds **30**, **31** were prenylated flavonoids (Fig. 4). Taken together, these results suggest that compound **30** would be the best candidate for use as a cosmetic agent.

## Conclusion

In this paper, the authors have reviewed some of their previous studies on the search for bioactive substances from unexplored plants, including those from Egypt, Madagascar, and Okinawa. The chemical structures of the compounds obtained from these plants are highly diverse. It is hoped that further exploration of compounds useful to mankind will lead to the discovery of new drugs.
